# Event and Comment

**Published:** 1915-03

**Authors:** 


### DENTAL REGISTER

Vol. LXIX MARCH 1915 No. 3

### EVENT AND COMMENT.

WHAT SHALL BE THE CONTENT OF NEW DENTAL CURRICULUM? Now that both Faculties Associations have decided to require a four-year course for graduation, the question as to what shall be taught in the extended course, in order that the student shall get the most for his time and money, and the people have the degree of skill and training that our new social conditions seem to demand, must be decided.

Some instructors are contending for more time for the technical subjects, others for the scientific subjects, and some for a curriculum that will permit the dental graduate to secure his medical degree by one or two additional years in medicine. A considerable number of the most thoughtful believe the best use to which the added year may be put, is to spend it in the literary or scientific departments of a standard university in getting a more thorough grounding in the scientific subjects which form the basis of all medical science instruction. Such subjects as general biology, physics, and chemistry have been most frequently suggested.

The contention of those demanding an extension of the technical branches is that there is at present a lack of thoroughness in the technical training, because the field has broadened so much faster than the colleges have advanced their teaching capacities that the college product of today, so far as available technical skill is concerned, is inferior to that of twenty-five years ago. It would be an herculean task to prove or disprove this assertion, and so we are perhaps safer in concurring in the popular sentiment of the day, and plan to move more cautiously and conservatively, for the time at least.

The claim can safely be made, we believe, that there never has been so much nor so high an order of technical training attempted as at present, and it may be that because of our efforts to teach so many more technical subjects we fail to exact from our students the degree of skill formerly secured when fewer and less complicated technical subjects were attempted. Teachers of experience, for a number of years, maintain that we are laying the foundations for the acquirement of a wider technical training, if not for higher technical skill, better today than ever before. Recent graduates of today are attempting technical practices that older practitioners, if they undertake them at all, do so only after special post-graduate instruction. We are of the opinion that with our present tendencies to the development of higher skill through the post-graduate schools, dental society clinics, and better fraternal relations, the complete technical education of the dentist should not be expected in the completed dental course. Shall we not rather plan to give a course that proposes to lay the foundations broad enough and deep enough to leave to the individual in large measure that part of his training which from the very nature of the case is peculiarly personal. A technical training, however complete, that has only a little scientific back-ground, and a small artistic perspective in the way of ideals, is likely to be perfunctory in practice and disappointing in results. If this be true then it would seem the part of wisdom to use the new opportunity to improve the student's capacity for acquiring the ideal in a technical education, rather than to use the time to hand over to him a ready-to-use training, which may perhaps need to be reworked by him after leaving college, and under adverse circumstances, before he feels sufficiently at home with it to put it to practical use with any degree of success to his patients. In plain words, isn't it more necessary at this stage of our development to train our students to think for themselves, correctly, and to more definite purposes, than to handicap them with mechanical formulae which they learn from demonstrations or memorize from text or didactic instruction, in which they have never had the pleasure of mental conception or spiritual insight? We are not suggesting here a broad cultural training, much as we feel its need, but a reasonable emphasis on the development of that in the dental student which shall make him act upon his own initiative, and which shall supply him with a basis for correct thinking and acting, not only as a student in college, but after he has taken up the active work of serving his patients. It is a common remark that dentists do not love or read their literature; and the reason is that the literature offers little inducement. How can we expect a type of literature that is better than its producers! How shall we induce dentists to read until they know how to write themselves, and to enjoy what others write? It seems clear to us that we must get back to the fundamentals. We must start right.

The average high-school graduate from the best high-schools in the land is not ready to appreciate the professional curriculum as it is at present administered. He comes from a high-school where he is taught to memorize pages from a text-book, and to recite the contents the day following, immediately to forget it, as it does not appeal to his sense or reason; consequently his whole course in high-school has done little more for him than to quicken his memory. He gets his instruction in a very different form when he enters college. He is expected to be vitally interested in each subject he takes, as each one is planned to prepare him to work out for himself a successful career. But owing to a faulty high-school training he fails to meet the new method of instruction. He can't memorize Gray's Anatomy, or any other text-book that is put into his hands. He hasn't been taught to listen to a lecture, to take notes, or to appropriate the fine points which the teachers labor to put into his thinker. He finds himself at sea, gets discouraged, then reckless, and too often falls into the habit of depending upon his wits to secure his credits. At the end of the first year he has made little or no progress and perhaps is morally unfit to proceed with his professional training. This is not always the case, but it is manifestly true in so many instances, and there is abundant reason to feel that it is substantially the case with so many students, that it seems there should be a remedy for it.

Our observation is that the average high-school is preparing its students for vocations, rather than for the professions. The need therefore seems to be for an intermediate training that shall equip high-school graduates to take up professional studies to advantage. Just what this should be we are not here prepared to discuss. It might be a preparatory course in a special school; or, as the medical profession has decided, two years of special work in a literary college. If our dental schools were prepared to give such courses it might be the wisest possible use of the extra year to devote it largely to cultural studies that would make real students of our matriculates.

Such scientific and philosophical studies as could be advantageously used in this course would serve to quicken the student's interest or help to decide that he had made an improper selection for a life work, and should enable him to change to some field where his capabilities would be more readily developed. We believe our best interests will be secured by requiring at least one year of specified work in a literary school before taking up the special work of the technical course. If this is impracticable, some provision must be made in the dental curriculum for these cultural subjects, or all instructors in the technical courses must introduce into their instruction the spirit of research, and strive to develop a capacity and desire for consecutive and productive thinking.

